# Variability of salivary analytes under daily conditions and their implications for periodontitis biomarkers

**DOI:** 10.3389/fdmed.2024.1369186

**Published:** 2024-03-18

**Authors:** Amanda Carolina Souza Delfino Rocha, Renata Klemp Orlandini, Ana Carolina Fragoso Motta, Juliana Barchelli Pinheiro, Gilberto André e Silva, Viviane de Cássia Oliveira, Alan Grupioni Lourenço

**Affiliations:** ^1^ Department of Basic and Oral Biology, Ribeirão Preto School of Dentistry, University of São Paulo, Ribeirão Preto, Brazil; ^2^ Department of Stomatology, Public Oral Health, and Forensic Dentistry, Ribeirão Preto School of Dentistry, University of São Paulo, Ribeirão Preto, Brazil; ^3^ Department of Dental Materials and Prosthesis, Ribeirão Preto School of Dentistry, University of São Paulo, Ribeirão Preto, Brazil

**Keywords:** biomarkers, saliva, periodontitis, repeated measurements, variability, IL-6, IL-8, total protein

## Abstract

**Introduction:**

Recent studies have identified inflammatory mediators as potential biomarkers for monitoring or diagnosing periodontitis. However, the brief half-life of these mediators, coupled with their variability among different individuals and across different stages of periodontal disease, may limit their reliability as biomarkers.

**Methods:**

In this study, we assessed the concentration profile of salivary biomarkers (IL-6, IL-8, and total protein) through repeated measurements within the same day and across different days in 79 patients exhibiting various states of periodontal health: intact periodontium, stable periodontitis, and active periodontitis. Additionally, we explored how daily variations, such as the interval between toothbrushing and eating, impact the levels of these salivary biomarkers and their diagnostic efficacy for periodontitis activity.

**Results:**

Our results showed high salivary levels of IL-6 and total proteins in periodontitis patients (*p* < 0.001), with detection ability reflected by an Area Under the Receiver Operating Characteristic Curve (AUC-ROC) ranging between 0.709 and 0.852. Conversely, IL-8 levels were higher in patients with intact periodontium (*p* < 0.001), with an AUC-ROC for periodontitis detection between 0.671 and 0.815. Daily activities such as toothbrushing and eating influenced the levels of specific analytes, particularly total proteins (*p* < 0.001), but this did not affect their ability to detect periodontal disease activity. The highest measurement agreement, assessed by Intraclass Correlation Coefficients (ICC), was found for IL-6, with no significant differences in agreement between same-day and different-day measurements.

**Conclusions:**

Our study demonstrated consistency in the repeated measurements of salivary analytes, both within the same day and across different days, except for salivary total protein levels. These analytes exhibited variability within a range that did not undermine their effectiveness as biomarkers for periodontal disease.

## 1 Introduction

Periodontitis, a prevalent chronic inflammatory disease affecting the tissues supporting teeth, poses a major challenge to global oral health. The WHO's 2022 report indicates that nearly 19% of the global population suffers from this condition (1). Despite technological advances, the diagnosis of periodontitis still primarily depends on evaluating tissue damage, a method that falls short in accurately representing the current state of disease activity (2, 3). In this context, salivary biomarkers are increasingly recognized as a promising approach for improving the diagnosis and monitoring of periodontitis (4).

Saliva is an ideal medium for biomarker discovery due to its non-invasive, safe, and simple collection process, which facilitates repeated sampling with minimal discomfort to the patient (5). Inflammatory mediators, such as Interleukin 6 (IL-6) and Interleukin 8 (IL-8), along with total salivary proteins, are gaining attention as salivary biomarkers because they are closely related to the progression of periodontal disease (2, 6–8). This interest is based on several studies that have reported elevated levels of these analytes in the saliva of patients with periodontal disease, highlighting their potential usefulness as diagnostic indicators or markers of disease progression (5, 9, 10).

Interleukin-6 (IL-6) plays a pivotal role in periodontal disease, not only because it is intricately involved in the bone resorption process characteristic of periodontitis but also as a regulator of acute phase proteins in inflammation (9, 11). Similarly, Interleukin-8 (IL-8) is crucial for the innate immune response to microorganisms in dental biofilm, facilitating neutrophil recruitment and angiogenesis (6, 12). Additionally, the total protein content in saliva is gaining recognition as a promising biomarker. It reflects the overall levels of inflammation and may serve as an indicator of periodontal health status (4, 13).

While these analytes demonstrate a strong association with the progression of periodontal disease, various factors introduce challenges that may limit their utility as biomarkers. These challenges include their short half-life, the variability of concentrations within the same individual, and their fluctuating levels corresponding to different stages of periodontal disease (14, 15). Studies indicate that salivary levels of these markers can vary significantly in measurements taken on different days in the same individual (5, 16). This variability is influenced by numerous factors, such as sympathetic and parasympathetic nervous system activity, medication usage, circadian rhythms, and physical exercise (17–19). Moreover, while some research presents conflicting findings, evidence suggests that hormonal fluctuations associated with puberty, pregnancy, menstrual cycles, and related disorders may also affect these biomarker levels (16, 20–23).

Additionally, everyday factors such as nutrition, hydration, and oral hygiene can influence the variability of salivary analytes (5, 24). To address this variability, numerous studies have standardized saliva collection methods by setting specific times for collection and controlling for the period since the last tooth brushing and meal (7, 8, 25). However, even with stringent saliva collection criteria, sample variability remains a significant challenge. For example, common guidelines for saliva collection typically involve abstaining from eating and brushing teeth for at least an hour prior to collection (2, 3, 7, 8, 11, 16, 18). This, however, results in a wide range of sample variations, from individuals who adhere to the minimum requirements to those who haven’t eaten or brushed their teeth in the past 12 h. Such disparities raise concerns about the heterogeneity of the samples collected, especially in the context of researching salivary inflammatory markers, where the impact of common daily variations on their concentration levels is not fully understood.

The precision of a biomarker is defined by its measurement reproducibility, meaning that identical results should be consistently obtained when the same test is repeated under identical conditions (26). Furthermore, effective salivary biomarkers should more significantly reflect changes due to periodontal disease rather than daily variations in saliva composition. However, studies focusing on the variability in total salivary protein concentrations are limited. To our knowledge, no studies have examined the impact of daily variables, such as intervals between tooth brushing and eating, on the salivary levels of IL-6 and IL-8. Moreover, the influence of these variables on the diagnostic capability of these biomarkers remains unexplored.

For this reason, the primary objective of this study was to evaluate the concentration profile of salivary biomarkers by performing repeated measurements within a single day and across different days, in patients with different states of periodontal health: intact periodontitis, stable periodontitis and active periodontitis, in order to assess the diagnostic capacity of this biomarkers in distinguishing cases of periodontitis from those of intact periodontium and stable periodontitis. Additionally, this investigation explored the impact of common daily variations, such as the interval between toothbrushing and eating, on the levels of these salivary biomarkers and their ability in diagnosing periodontitis activity.

## 2 Materials and methods

### 2.1 Study population and design

This study was conducted from March 2020 to February 2022. All participants were fully informed about the research objectives and provided informed and voluntary consent. The project received approval from the Research Ethics Committee of FORP (CAAE 98638818.2.0000.5419). Participants were categorized based on the new classification system for periodontal disease by the American Academy and the European Federation of Periodontology (27) into three groups: (1) Intact Periodontium Group (IP group): Individuals exhibiting no periodontal attachment loss, a probing depth of up to 3 mm, bleeding on probing in less than 10% of the sites; (2) Stable Periodontitis Group (SP group): Participants with attachment loss, a probing depth of up to 4 mm, no sites with probing depth ≥4 mm with bleeding, and bleeding on probing in less than 10% of the sites; and (3) Periodontitis Group (P group): Patients showing detectable proximal attachment loss on two or more non-adjacent teeth and/or attachment loss ≥3 mm on the buccal or lingual/palatal surfaces in at least 2 teeth due to periodontal disease. These individuals also exhibited a probing depth ≥5 mm or a probing depth ≥4 mm with bleeding on probing.

The number of participants was calculated using G*Power 3.1 software, with an effect size of 20%. The sample size calculation was based on achieving a statistical power of 80% and a significance level of 0.05. This resulted in a total of 18 participants per group for the study.

All participants were selected based on the following inclusion criteria: (1) Absence of systemic conditions that could affect salivary cytokine levels, such as diabetes mellitus, antineoplastic treatment, or immunosuppression; (2) No use of anti-inflammatory or antibiotic medications in the past three months; (3) No periodontal treatment received within the last 6 months; and (4) Have more than 14 teeth.

### 2.2 Study design and Saliva collection

The duration of individual participation in the study extended over 16 days, involving three visits. In the initial visit (Day 0), a calibrated researcher performed a periodontal examination of the entire mouth using a North Carolina millimeter periodontal probe (HuFriedy, Chicago, IL). The evaluated parameters included the Gingival Bleeding Index (BI), Probing Depth (PD), and Clinical Attachment Level (CAL) at six different sites for each tooth. The BI was determined by the presence or absence of bleeding within 20 s of probing. PD was measured from the gingival margin to the base of the sulcus or periodontal pocket, while CAL was assessed from the cementoenamel junction to the most apical portion of the sulcus or pocket.

The day following the periodontal examination (Day 1), three saliva collections were conducted to introduce variability in relation to the time elapsed since the last oral hygiene and feeding session.

**Figure 1:**
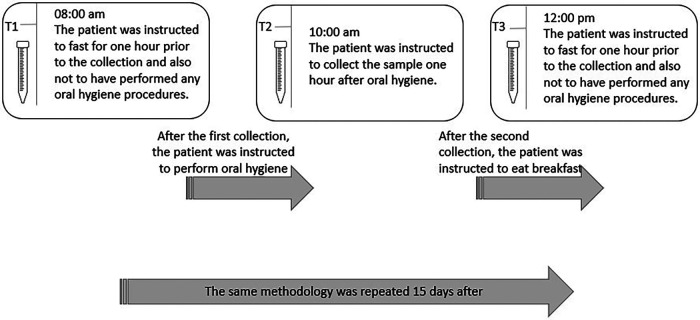
Description of the saliva collection methodology.

The first saliva collection (T1) occurred at 8 am, with participants in a fasting state and having not performed morning tooth brushing, ensuring more than 6 h since the last oral hygiene and eating. Immediately following T1, participants were instructed to perform oral hygiene. Approximately one-hour post-tooth brushing, around 9 am, the second collection (T2) was conducted. Participants were still fasting, guaranteeing more than 7 h without food and 1 h after the last oral hygiene. Immediately following T2, participants were instructed to have breakfast. The third collection (T3) took place approximately two hours after breakfast, around 11 am, corresponding to 2 h post-meal and 3 h after the last oral hygiene. The same methodology for saliva collection was repeated 15 days later (Day 16). Given the significant impact of hormonal fluctuations on salivary composition, setting a 15-day interval between saliva collections is strategically justified. This timing ensures that the collections span the transitions between the different phases of the menstrual cycle in female participants: the follicular phase (days 1–14), the luteal phase (days 15–28), and the menstrual phase (21). The saliva collection methodology is illustrated in Figure 1.

Importantly, all saliva collections adhered to standard instructions of abstaining from eating, drinking, smoking, or performing oral hygiene for at least one hour before each collection. This approach was designed to introduce a broad range of variability in collection conditions while still adhering to the standard guidelines commonly mentioned in scientific literature.

Saliva collection from all participants involved instructing them to rinse with tap water. The non-stimulated saliva was collected according to the method described by Navazesh et al. (25). Subsequently, saliva samples were collected on ice, and aliquots were prepared, which were then frozen at −80°C until the analysis phase.

### 2.3 Cytokine analysis

For the determination of protein concentration, we used the Bio-Rad DC Protein Assay (DC™ Protein Assay Reagents Package) colorimetric assay, following the manufacturer's guidelines.

To quantify salivary levels of IL-6 and IL-8, sandwich ELISA kits provided by Peprotech (California, USA) were used. Absorbance measurements at 405 nm were recorded using a Thermo Scientific Multiskan GO ELISA reader, in accordance with the manufacturer's instructions.

### 2.4 Statistical analyses

Statistical analyses were performed using SPSS software version 25.0 (IBM Corp, Armonk, NY, USA), and graphs were generated using GraphPad Prism 8.0 (GraphPad Software, Boston, MA, USA). A two-tailed test was adopted with an alpha of 0.05. Descriptive analyses included the calculation of means, standard deviations, medians, and quartiles. The chi-square test was utilized to assess qualitative independence. The normality of data distribution was evaluated using the Shapiro–Wilk test. For demographic data, one-way ANOVA was applied to variables with a normal distribution, while the Kruskal–Wallis test was used for non-normal variables.

Analysis of the dependent variables IL-6, IL-8, and Total Protein employed a Linear Mixed Model, with participants as a random factor. Fixed factors included group (periodontal status), time of study (T1, T2, and T3), and collection days (D1 and D16), using an AR (1) covariance matrix. The choice of an AR (1) covariance matrix to model the temporal correlation among repeated measurements for each participant was informed by an analysis based on the Akaike Information Criterion (AIC). Pairwise comparisons were adjusted using the Bonferroni method. The effect size was quantified by calculating the mean difference (MD) for each comparison between fixed factors, as well as within each fixed factor, accompanied by the respective 95% confidence intervals (CIs).

Intraclass Correlation Coefficients (ICC) were calculated using a one-way random-effects model to evaluate consistency between salivary analyte measurements within the same day (T1, T2, and T3) and on different days (D1 and D16). Following Cicchetti, (28), ICCs were classified as: poor (<0.40), fair (0.40–0.59), good (0.60–0.74), and excellent (0.75–1.00).

For assessing periodontal disease activity, we utilized the Area Under the Curve (AUC) of the Receiver Operating Characteristic (ROC). Participants were categorized into “periodontal health” (21 with intact periodontium and 22 with stable periodontitis) and “active disease” (36 with active periodontitis). In the discriminant analysis between periodontitis and intact periodontium, stable periodontitis cases were excluded. The predictive capacity of salivary cytokine levels was assessed by AUC-ROC, considered significant if above 0.5. For AUC-ROC analysis, all cytokine samples (T1–3) were examined collectively, and comparative analyses were conducted for each collection time (T1, T2, and T3) to identify the most opportune moment for optimizing diagnostic accuracy.

## 3 Results

**Figure 2:**
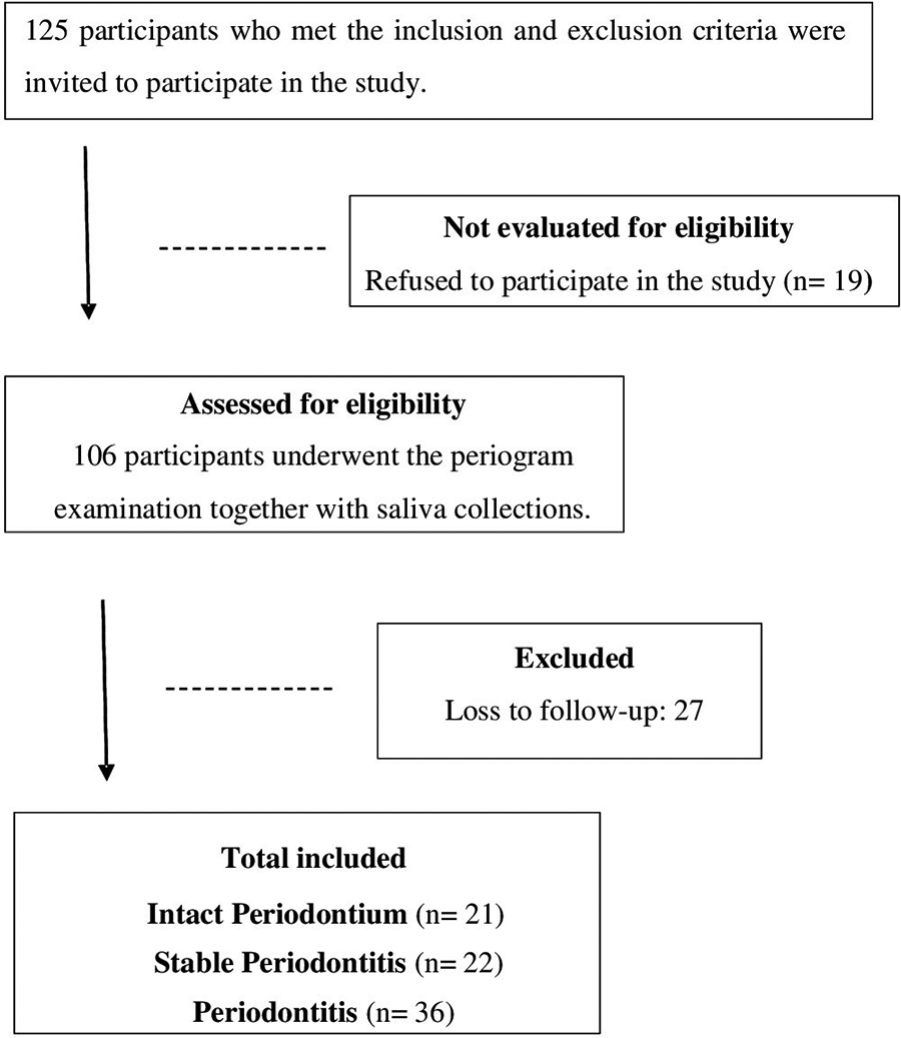
Flowchart of study design.

This cohort study included 21 participants in IP Group, 22 in SP Group, and 36 in the P Group. The participant inclusion process is depicted in the flowchart in Figure 2.

An analysis of demographic and clinical data indicated a homogeneous distribution across the groups in terms of variables such as gender, smoking habits, drug use, alcohol consumption, presence of chronic systemic diseases, and medication usage. The sociodemographic and clinical characteristics of the participants are detailed in Table 1. All patients diagnosed with periodontitis were classified as being in either stage III or IV of the disease.

**Table 1:** Sociodemographic and clinical profile of the participants.

		IP group	SP group	P group	*P* value
No. of participants		21	22	36	
Gender	Men	8 (38.1%)	8 (36.4%)	14 (38.9%)	0.982
	Women	13 (61.9%)	14 (63.6%)	22 (61.1%)	
Age (years)		39 (±8)	52,0 (±11.5)	59,5 (±14.3)	<.001
Ethnicity	White	19 (90.5%)	16 (72.7%)	31 (86.1%)	0.2492
	Non-white	2 (9.5%)	6 (27.3%)	5 (13.9%)	
Education	Elementary school	2 (9.5%)	2 (9.1%)	12 (33.3%)	0.004
	Middle school	0 (0.0%)	1 (4.5%)	4 (11.1%)	
	High school	5 (23.8%)	12 (54%)	10 (27.8%)	
	College	14 (66.7%)	3 (13.6%)	7 (19.4%)	
Smoking	Yes	2 (9.5%)	2 (9.1%)	5 (13.9%)	0.815
	No	19 (90.5%)	20 (90.9%)	31 (86.1%)	
Drug addiction	Yes	0 (0.0%)	0 (0.0%)	1 (2.8%)	0.546
	No	21 (100%)	22 (100%)	35 (97.2%)	
Alcohol consumption	Yes	6 (28.6%)	5 (22.7%)	8 (22.2%)	0.851
	No	15 (71.4%)	17 (77.3%)	28 (77.8%)	
Comorbidities	No comorbidities	13	11	16	0.816
	Neurological	4	3	4	
	Cardiovascular	4	6	12	
	Endocrine	3	5	6	
	Respiratory	0	0	1	
	Musculoskeletal	0	0	1	
	Autoimmune	0	0	2	
Medication	Yes	10 (47.6%)	11 (50%)	18 (50%)	0.983
	No	11 (52.4%)	11 (50%)	18 (50%)	
No. of teeth		28 (±4)	27 (±3)	25 (±5)	<.001
PD sites ≥4 mm		0 (±0.0)	0.50 (±2.0)	11 (±27.0)	<.001
CAL sites ≥4 mm		0 (±0.0)	14 (±17.8)	31.5 (±51.0)	<.001
BI (%)		1.7 (±3.5)	1.65 (±4.87)	24.4 (±29.9)	<.001
PI (%)		0.6 (±4.46)	0.0 (±3.74)	20,5 (±40.2)	<.001
DMFT		6 (±13.0)	10 (±15)	12 (±9.75)	0.262

IP Group, intact periodontium group; SP group, stable periodontitis group; P group, periodontitis group; N^o^, number; BI, gingival bleeding index; PD, probing depth; CAL, clinical attachment level; PI, plaque index; DMFT, decayed, missing and filled teeth.

### 3.1 Analysis of salivary levels of IL-6, IL-8, and total protein in different periodontal health conditions and their variability across study times

**Figure 3:**
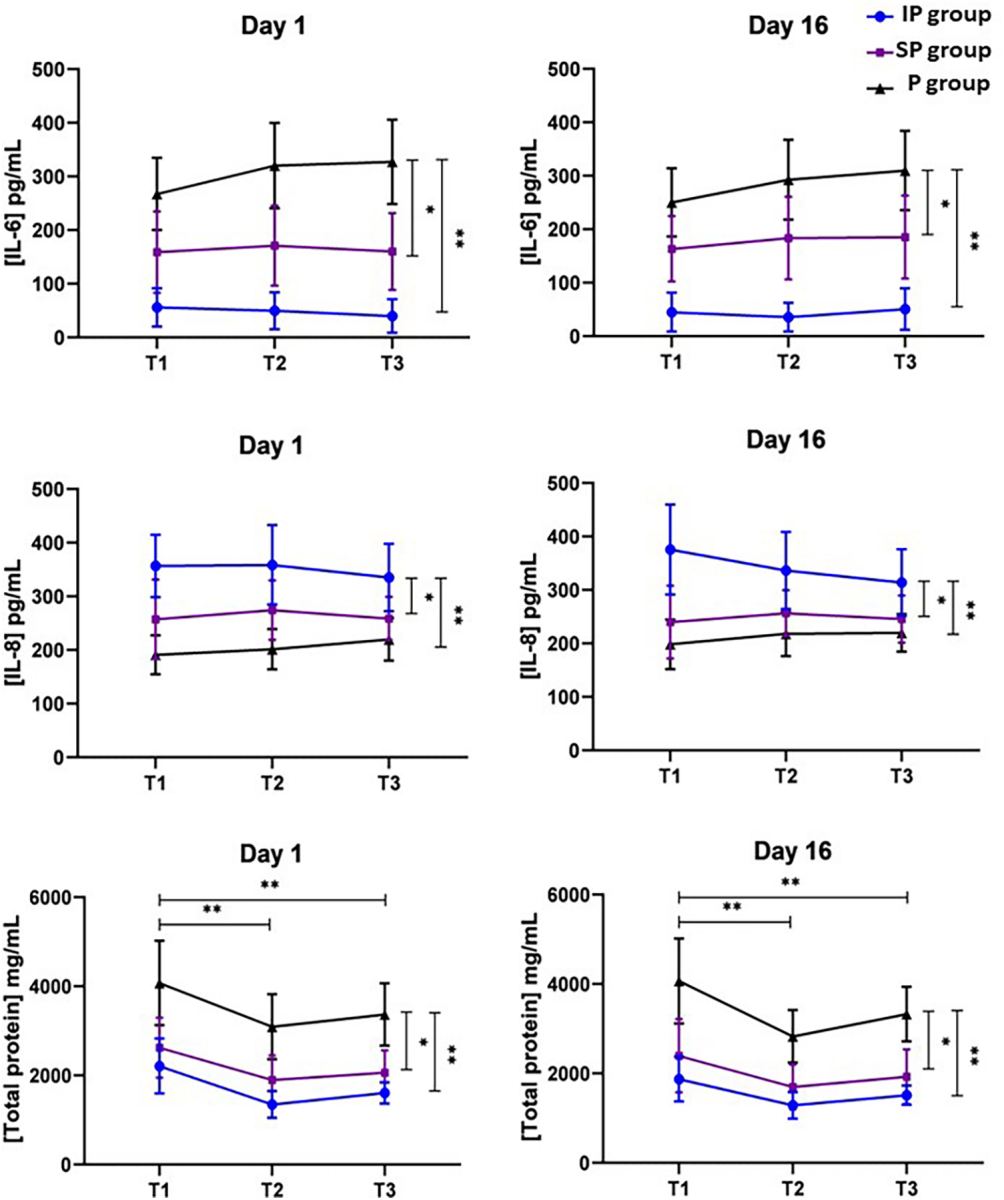
Profiles of salivary biomarker concentrations through repeated measurements within a single day (T1, T2, and T3) and across different days (Day 1 and Day 16) in patients with intact periodontium (IP group), stable periodontitis (SP group), and periodontitis (P group). T1: Saliva collection from participants in a fasting state, without having performed morning tooth brushing. T2: Saliva collection from participants who are still fasting and 1 h post-last oral hygiene. T3: Saliva collection occurring two hours after breakfast and 3 h following the last oral hygiene. *p < 0.05; **p < 0.001.

The salivary levels of IL-6 and total protein were significantly higher in the P group compared to both the IP group (*p* < 0.001) and the SP group (IL-6: *p* = 0.013; Total protein: *p* = 0.002). Specifically, IL-6 levels in the P group were higher by a mean difference (MD) of 248 pg/ml compared to the IP group, with a confidence interval (CI) of 29–219 (*p* < 0.001), and by 124 pg/ml compared to the SP group (CI: 152–345; *p* = 0.013). For total protein, the P group exhibited a significant increase, with an MD of 1,821 pg/ml over the IP group (CI = 910–2,732; *p* < 0.001) and 1,360 mg/ml over the SP group (CI: 910–2,732 mg/ml; *p* = 0.002). Conversely, IL-8 levels were higher in the IP group than in both the P group (MD = 138 pg/ml, CI: 195–81, *p* < 0.001) and the SP group (MD = 91 pg/ml, CI: 27–154, *p* = 0.002), as detailed in Figure 3.

In our investigation of the variation in salivary biomarker levels at different collection times within the same day, distinct patterns emerged in relation to the time elapsed since the last oral hygiene and feeding session. The levels of Interleukin-6 (IL-6) did not show significant changes across different times (*p* = 0.418), a pattern similarly observed in the levels of Interleukin-8 (IL-8), which remained consistent (*p* = 0.803). However, an influence of collection time was detected on total protein levels. The highest levels were recorded at T1, during the fasting period and prior to morning toothbrushing. Subsequently, there was a significant decrease in these levels one hour after toothbrushing (T2; MD = −849 mg/ml, CI: −1,253 to 445, *p* < 0.001) and two hours post-breakfast (T3; MD = −573 mg/ml, CI: −1,112 to −34, *p* < 0.001).

In comparing the data obtained on the two different collection days, Day 1 and Day 16, no significant variations were observed in the levels of IL-6 (MD = 4 pg/ml, CI: −10 to 17, *p* = 0.582), IL-8 (MD = 5 pg/ml, CI = −9 to 20, *p* = 0.453), and total protein (MD = 151 mg/ml; CI: −10 to 313, *p* = 0.07). This consistency in biomarker levels across different days is illustrated in Figure 3. Such findings suggest a notable stability in the levels of these biomarkers over time.

In terms of agreement in repeated measurements, assessed by ICC, salivary IL-6 levels exhibited greater consistency between measurements compared to IL-8 and Total Protein. Following the classification system by Cicchetti et al. (28), the ICC for IL-6 was categorized as “excellent”, whereas the ICCs for IL-8 and Total Protein were categorized as “good”. There were no significant differences in the ICCs between measurements taken on the same day and those taken on different days. This indicates a stable presence of these salivary markers over time, as shown in Table 2.

**Table 2:** Intraclass correlation coefficients and their confidence intervals for repeated measurements within a single day (Day 1 and Day 16) and across different days (between Day 1 and 16).

	IL6	IL8	Total protein
Day 1	0.896 (0.862–0.924)	0.722 (0.644–0.790)	0.698 (0.616–0.771
Day 16	0.898 (0.864–0.926)	0.717 (0.638–0.786)	0.637 (0.544–0.721)
Between day 1 and 16	0.861 (0.824–0.895)	0.664 (0.594–0.732	0.688 (0.620–0.753)

### 3.2 Study of diagnostic capacity for detecting periodontitis at different collection times

To assess the diagnostic accuracy in detecting periodontitis using ROC curves, two distinct analyses were conducted: Situation 1: This analysis examined the ability to differentiate cases of periodontitis from those with intact periodontium and stable periodontitis (periodontal health); Situation 2: This analysis focused on distinguishing periodontitis cases from those with intact periodontium. Additionally, each study time (T1, T2, and T3) was individually considered, along with the calculation of the AUC for all study times combined (–3).

In both situations, IL-6 demonstrated a larger AUC-ROC compared to the salivary levels of IL-8 and Total Protein, with the exception of T2 in Situation 1.

For none of the biomarkers did we observe any influence of the post-brushing and feeding interval on their AUC-ROC in detecting periodontitis. That is, the AUCs evaluated collectively (T1–3) were comparable to those evaluated at individual times (T1, T2, and T3). The AUC-ROC values were consistent across all study times for the evaluated biomarkers, as detailed in Table 3.

**Table 3:** Area under the ROC curve for examining the ability to differentiate cases of periodontitis from those with intact periodontium and stable periodontitis (situation 1), and the ability to distinguish periodontitis cases from those with intact periodontium (situation 2). Additionally, each study time (T1, T2, and T3) was individually considered, along with the calculation of the AUC for all study times combined (T1–3).

		IL-6	IL-8	Total protein
Situation 1	T1	0.731 (0.649–0.813)	0.707 (0.627–0.787)	0.709 (0.628–0.790)
	T2	0.747 (0.668–0.826)	0.719 (0.638–0.800)	0.763 (0.689–0.836)
	T3	0.771 (0.695–0.847)	0.671 (0.586–0.755)	0.766 (0.690–0.843)
	T1–T3	0.752 (0.707–0.797)	0.697 (0.650–0.744)	0.739 (0.695–0.784)
Situation 2	T1	0.818 (0.742–0.895)	0.815 (0.736–0.895)	0.745 (0.655–0.835)
	T2	0.833 (0.760–0.906)	0.769 (0.672–0.866)	0.815 (0.738–0.891)
	T3	0.852 (0.782–0.922)	0.733 (0.639–0.827)	0.804 (0.725–0.883)
	T1–T3	0.838 (0.796–0.879)	0.773 (0.720–0.825)	0.780 (0.732–0.828)

T1: Saliva collection from participants in a fasting state, without having performed morning tooth brushing. T2: Saliva collection from participants who are still fasting and 1 h post-last oral hygiene. T3: Saliva collection occurring two hours after breakfast and 3 h following the last oral hygiene. T1–3: Calculation of the AUC for all study times combined (T1–3).

Additionally, the AUC-ROC was consistently higher in Situation 2 compared to Situation 1 across all times analyzed. This suggests that distinguishing cases of periodontitis from intact periodontium cases was more effective than differentiating periodontitis cases from a combination of stable periodontitis and intact periodontium.

Despite notable variability in salivary total protein levels due to everyday conditions, this variation did not impact diagnostic accuracy, as indicated by the stability of the total protein AUC-ROC, as shown in Table 3.

## 4 Discussion

This study has identified variability in the measurements of potential salivary biomarkers due to daily challenges. Despite this variability, the biomarkers demonstrated satisfactory diagnostic accuracy. The importance of examining variability in repeated measurements lies in the necessity for measurement reproducibility: the same test, when applied to an unchanged patient, should yield consistent results (26, 29). Our findings indicate that while daily conditions can influence protein salivary concentrations, this variability does not impair the diagnostic accuracy for detecting periodontitis.

Remarkably, our study did not conduct strictly repeated measurements, as each collection was performed under unique hygiene conditions, food intake, and varying times. Nonetheless, it is desirable that variations in the salivary levels of these biomarkers should be predominantly influenced by periodontal disease rather than by daily variables such as post-brushing and feeding times (26).

Our study noted that IL-6 concentrations are higher in the saliva of patients with periodontal disease, and these elevated levels are capable of detecting active periodontitis. This observation is in line with other research findings, where IL-6 has demonstrated the ability to detect periodontitis with a sensitivity ranging from 52% to 80% and a specificity between 48% and 87% (30). Ebersole et al. (7) also reported findings consistent with our study, identifying significantly higher concentrations of salivary IL-6 in periodontitis patients compared to those with gingivitis or in a periodontally healthy state.

In terms of IL-8, our study identified elevated levels in patients with intact periodontium, aligning with the findings of Khalaf et al. (31). Similarly, Miranda et al. (32) observed high levels of IL-8 in the gingival crevicular fluid of healthy sites in contrast to sites affected by periodontitis. However, the link between salivary IL-8 levels and periodontitis remains unclear. Studies such as those by Teles et al. (33) and Lisa Cheng et al. (34) did not establish a direct correlation between salivary IL-8 levels and the incidence of periodontal disease.

The systematic review by Finoti et al. (35) underscores the variability and lack of consensus in research examining salivary IL-8 levels in periodontitis patients. The authors suggest that discrepancies in findings may be attributed to factors such as differing methods of saliva collection and processing, biomarker quantification techniques, and varying stages of periodontitis.

The findings from our study regarding total salivary protein align with the results reported by Cui et al. (36) and Kejriwal et al. (37), who also observed an increase in total protein concentration in the saliva of periodontitis patients compared to healthy individuals. This consistent correlation underscores the potential of salivary total protein as a biomarker for periodontal disease.

To our knowledge, this study is the first to evaluate the variations in salivary pro-inflammatory cytokines IL-6 and IL-8 in response to daily challenges and to assess their correlation with the diagnostic capacity for periodontitis. Concerning total protein, our results align with those of Justino et al. (38), who observed a decrease in salivary total protein levels following tooth brushing. The finding that daily conditions can significantly affect salivary total protein levels is noteworthy, particularly considering that many studies use these levels as an endogenous control for normalizing and correcting experimental biases in their analytes (39, 40). Our findings suggest that the use of salivary total protein as a normalization factor in experimental settings should be approached with caution due to its variability in response to common daily activities.

We found no significant differences in the agreements of repeated analyses, as measured by ICC, whether conducted on the same day or on different days. These results indicate the temporal stability of the analytes. Although studies suggest that hormonal variations may affect periodontal tissue metabolism (22, 23), our study did not observe any significant gender-based discrepancies in salivary analyte levels (results not shown). Notably, the 15-day interval between collections was chosen to accommodate potential changes in the menstrual cycle phases of female participants (16). Nevertheless, these hormonal variations did not contribute to the variability of the analytes across different days, a finding also observed by other studies that did not notice the influence of the menstrual cycle on the levels of salivary analytes (41).

In 2009, Thomas and colleagues (26) evaluated the ICC for IL-6 through repeated measurements over several days. They reported lower levels of agreement than those observed in our study, a difference that can be attributed to variations in methodology. In their study, Thomas and colleagues (26) carried out six different measurements, with some collections self-administered by patients at home. Such a setup could lead to increased variability between collections, potentially resulting in greater variability in the ICC values.

One limitation of our study is the variation in clinical and demographic profiles across different groups. Predominantly, the intact periodontium group consisted of younger patients with higher educational levels. Despite concerted efforts to balance participant ages, it proved challenging to recruit patients over 40 years old with intact periodontium. Another limitation relates to the control of participants’ hydration levels. While we instructed participants to refrain from consuming liquids one hour before each collection, as per Navazesh et al. (25), specific monitoring of hydration levels was not conducted. This is a significant consideration, given that hydration status can affect saliva viscosity, total protein concentrations, and osmolality (24).

## 5 Conclusion

In conclusion, our study has demonstrated that salivary levels of IL-6, IL-8, and total protein significantly vary among patient groups with differing periodontal conditions, affirming the discriminative potential of these biomarkers in distinguishing between healthy states and periodontal disease. Notably, the levels of IL-6 and IL-8 exhibited remarkable stability, unaffected by the timing of saliva collection, reinforcing the reliability of these markers under a range of temporal conditions. Although daily conditions, such as oral hygiene and diet, were found to influence fluctuations in total protein levels, these variations did not compromise its diagnostic value for detecting periodontitis. Therefore, the presence of daily variabilities and measurement fluctuations at different times did not diminish the predictive efficacy of these biomarkers for periodontal disease. Given the scarcity of research on the variability of salivary biomarkers within the context of periodontal disease, this area emerges as a crucial avenue for future investigation. Consequently, we underscore the critical need for further studies aimed at elucidating the variability of these salivary biomarkers and assessing their impact on diagnostic accuracy.

## Data Availability

The original contributions presented in the study are included in the article/Supplementary Material, further inquiries can be directed to the corresponding author.
