# Supplementary material for: Variability of salivary analytes under daily conditions and their implications for periodontitis biomarkers
Source: Front Dent Med. 2024 Mar 18;5:1369186. doi: 10.3389/fdmed.2024.1369186 (PMC11797852; doi:10.3389/fdmed.2024.1369186)
Supplement: Supplementary file 1 [file Datasheet2.docx]

| **colunn** | **Type variable** | **Definition** | **Categorical** |
| --- | --- | --- | --- |
| code | text | patient id |  |
| age | discrete | Age in years |  |
| ethnicity | categorical |  | 0 – white  1 – non white |
| gender | categorical |  | 0 – woman  1- man |
| study_group | categorical |  | 0 – intact periodontum   1. stable periodontis 2. periodontitis |
| stage_periodontitis | categorical |  | 0 – stage III   1. stage IV |
| education | categorical | patient education level | 0 – elementary scholl   1. middle school   2 – high school  3 - college |
| smoking | categorical |  | 0 – No  1 - Yes |
| alcohol | categorical | Alcohol consumption | 0 – No  1 - Yes |
| drug | categorical | Drug addiction | 0 – No  1 - Yes |
| comorbidities | categorical | comorbidity medical | 0 – no comorbidities  1 – neurological  2 – cardiovascular  3 – endocrine  4 - musculoskeletal  5 – autoimmune disease  6 – neurological and cardiovascular  7 – endocrine and cardiovascular  8 – neurological and endocrine  9 - cardiovascular and respiratory |
| medication | categorical |  | 0 – No  1 - Yes |
| bleeding_index | continuous | gengival bleeding index (percentage) |  |
| plaque_index | continuous | plaque index (percentage) |  |
| dmft | discrete | decayed, missing and filled teeth. |  |
| n_teeth | discrete | number of teeth |  |
| n_pdsites | discrete | number of probing depth sites ≥ 4mm |  |
| n_calsites | discrete | number of clinical attachment level sites ≥ 4mm |  |
| saliva_collection | categorical | times of the study | 1. T1 2. T2 3. T3 |
| day_colection | categorical | saliva collection on day 1 and day 16 | 1. Day 1 2. Day 16 |
| salivary_flow | continuous | salivary flow in mL/min |  |
| IL6 | continuous | salivary level of IL-6 in pg/mL |  |
| IL8 | continuous | salivary level of IL-8 in pg/mL |  |
| total_protein | continuous | salivary level of total protein mg/mL |  |
